# Omega-3 Adjunctive Therapy in Idiopathic SSNHL: A Randomised, Triple-Blind, Placebo-Controlled Trial

**DOI:** 10.22038/IJORL.2023.71955.3444

**Published:** 2023-11

**Authors:** Benyamin Rahmaty, Kayvan Aghazadeh, Sasan Dabiri, Masoud Motasaddi Zarandy, Ali Kouhi, Nasrin Yazdani, Reza Erfanian, Ardavan Tajdini, Saeed Sohrabpour, Fatemeh Safi, Reza Shamsa, Hamideh Ravand, Neda Jafari

**Affiliations:** 1 *Otorhinolaryngologist-Head and Neck Surgeon, Otorhinolaryngology Research Center, Amir Alam Hospital, Tehran University of Medical Sciences, Tehran, Iran.*; 2 *Department of Radiology, Arak University of Medical Sciences, Arak, Iran.*

**Keywords:** Omega-3, Sudden sensorineural hearing loss (SSNHL), Vertigo

## Abstract

**Introduction:**

Idiopathic Sudden Sensorineural Hearing Loss as a subset of sensorineural hearing loss will be confirmed by a progressive hearing loss of at least 30 dB at three contiguous frequencies over 72 hours or less. A sudden or abrupt hearing loss correlates with the time course, and a vascular event is presumptive aetiology. There is an inverse association between Omega-3 consumption and hearing loss. This study aimed to evaluate the efficacy of Omega-3 adjunctive therapy in Idiopathic Sudden Sensorineural Hearing Loss by audiometric assessments.

**Materials and Methods:**

In this randomised, triple-blind, placebo-controlled trial, all participants aged 18-70 with a history of sudden deafness (within 12 hours and ≤ 30 days) were eligible for enrollment. They were included if audiology diagnostic tests confirmed the SSNHL. Ultimately, they were randomised to the Omega-3 group and the placebo group.

**Results:**

Thirty-three patients were randomly allocated to the Omega-3 group and thirty-two to the placebo group. Vertigo (32.3% of all patients) and underlying conditions had significant relationships with complete response (C.R.)-final hearing level ≤of 25 dB in pure-tone average (P < 0.05). There was no significant difference between both groups before and after treatment. Although it was not statistically significant, patients in the Omega-3 group had faster recovery than placebo.

**Conclusions:**

Omega-3 adjunctive therapy did not have a therapeutic effect on SSNHL patients. Moreover, C.R. happened in half the patients. Vertigo and underlying conditions considerably worsen the recovery from SSNHL.

## Introduction

Idiopathic Sudden Sensorineural Hearing Loss (SSNHL), as a subset of sensorineural hearing loss (SNHL), will be confirmed after excluding other etiologies such as infectious (serous, viral, and bacterial labyrinthitis), neoplastic (brain or CP-angle), traumatic, ototoxic (aminoglycosides, chemothera- peutics), immunologic, vascular, and developmental idiopathic entities (1). 

The SSNHL usually develops as a progressive hearing loss of at least 30 dB at three contiguous frequencies over 72 hours or less (2). The most common presentation is noticing unilateral hearing loss (H.L.). Fluctuating H.L., a sensation of aural fullness, acute tinnitus, and vertigo or disequilibrium intercurrent to sudden deafness are other patients' complaints (1,3). This idiopathic syndrome has several principal theories of its probable pathogenic mechanisms. These theories include viral infection (viral neuritis or cochleitis), bacterial infections (subclinical meningoencephalitis), intracochlear membrane ruptures, immune-mediated inner ear disease, and vascular disorders (4). An intriguing hypothetical aetiology for idiopathic SSNHL is cochlear damage following vascular events or hemostatic abnormalities that may occur after the cochlear blood supply occlusion. 

A sudden or abrupt hearing loss correlates with the time course and a vascular event. Therefore, based on this presumptive aetiology—vascular event, alteration of the oxygenation of the Cochlea or perilymph may occur due to the changes in cochlear blood flow (4). Omega−3 as polyunsaturated fatty acids (PUFAs) — especially eicosapentaenoic acid (EPA) and docosahexaenoic acid (DHA) — and fish are protective against cardiovascular disease (5,6). There is also an inverse association between Omega-3 consumption and hearing loss (6). 

Based on the mentioned points, researchers of the current study hypothesised the following statements:

- Omega-3 improves speech discrimination score (SDS) or word recognition score (WRS) in patients with sudden sensorineural hearing loss (SSNHL)
- Omega-3 improves pure-tone average (PTA) in patients with sudden sensorineural hearing loss (SSNHL)
- Omega-3 improves speech reception threshold (SRT) in patients with sudden sensorineural hearing loss (SSNHL)

Researchers also raised a specific research question regarding Omega-3 adjunctive therapy in SSNHL:

- Which component(s) of audiology diagnostic tests may improve following Omega-3 adjunctive therapy in sudden sensorineural hearing loss (SSNHL) patients?

To tackle the mentioned hypotheses and research question, researchers included patients with idiopathic SSNHL after obtaining a thorough medical history and complete audiometric assessments.

## Materials and Methods

*TRIAL OVERSIGHT*

This Iranian study was a randomised, triple-blind, placebo-controlled trial to assess the efficacy of Omega-3 adjunctive therapy in Patients with idiopathic Sudden Sensorineural Hearing Loss (SSNHL). It was conducted at Amir Alam Hospital, where patients with SSNHL were recruited. This study was overseen by a steering committee—the Iranian Registry of Clinical Trials (IRCT). The Otorhinolaryngology Research Center of Tehran University of Medical Sciences also supported it. The funder did not affect the design or conducting of the trial and all steps, including data collection, analysis, manuscript writing, and submission decision. The trial protocol — available at the Iranian Registry of Clinical Trials (IRCT) — was approved by the Tehran University of Medical Sciences Ethics Committee. This trial was performed under the principles of the Declaration of Helsinki. The authors assume responsibility for the accuracy and completeness of the data and analyses, the trial’s accuracy, and this report to the protocol. The current study researchers also used the CONSORT reporting guidelines(7).

*PATIENTS*

Participants aged 18-70 with a recent SSNHL (≤ 30 days) were eligible for enrollment in the current study. Participants could be included when they had a recent history of sudden deafness or acute tinnitus intercurrent to aural fullness or sudden deafness within 12 hours. All patients underwent a thorough examination by two Otolaryngologists. History and clinical data were obtained, and microscopic and tuning fork tests were performed during a thorough otology examination. All participants who had a history of congenital deafness, brain tumours, anticoagulant medications, metabolic diseases (Refsum disease), inherited bleeding disorders, abnormal coagulation profile, corticosteroid contraindications, lipid-lowering drugs (intestinal agents, statins, niacin, and fibrates), lactation or pregnancy, and allergy to fish or fish oil were excluded. Ultimately, they were included if they were confirmed by audiology diagnostic tests (i.e., PTA, SRT, WRS, acoustic reflex threshold (ART), and tympanometry). All participants agreed and signed the informed consent form after explanations and clarifications. After completing the clinical data, patients were referred to the local Otorhinolaryngology Research Center in the hospital for randomisation and receiving their medication. 

*TRIAL PROCEDURES*

Participants were randomised in a 1:1 ratio to either the Omega-3 or the placebo groups. Randomisation was performed with the computer software Researchiser (Version 4.0) -from http://www.randomizer.org/. 

*Standard treatment:* All participants (Omega-3 and placebo groups) received their traditional medication—oral Prednisolone 1mg/kg/day (not to exceed 60mg/day) for a 10-day course in a single undivided dose. If PTA observed recovery at the end of ten days, extending the treatment for another 10 days was considered. This treatment was repeated until no further improvement was noted. After a full recovery, it was gradually tapered. 

**Fig 1:**
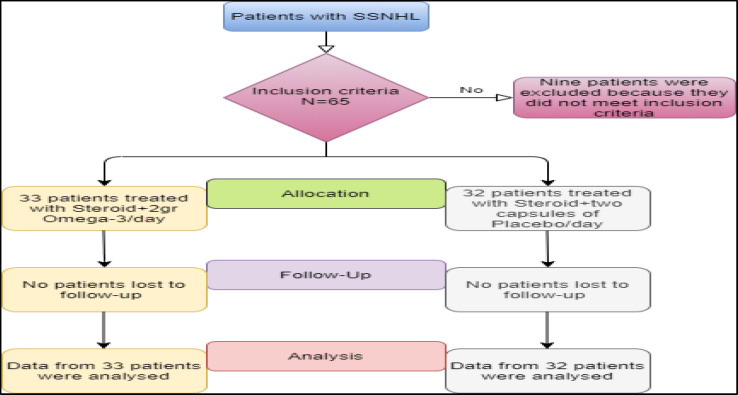
This flowchart represents the study protocol, allocation, follow-up, and analysis. Sixty-five patients were recruited, i.e., 33 in the Omega-3 group and 32 in the placebo group

If there was no recovery after any full-course oral treatment by Prednisolone, the second-line treatment was Intratympanic (I.T.) Dexamethasone 8 mg/ml, 0.5 CC per session/every other day/in total, for six sessions. All audiometry diagnostic tests were obtained following each 10-day course and repeated every ten days following a month after the initial treatment. Ultimately, the last audiometry tests were obtained after three months (0-10-20-30-90 days) (Figure 1).

**Fig 2:**
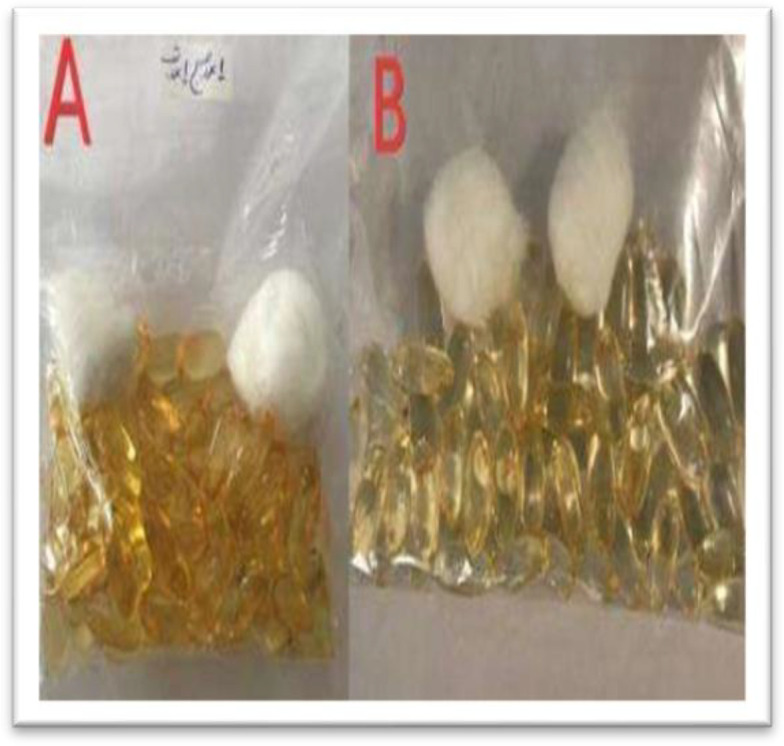
This Photo Represents Omega-3(A) And Placebo (B) Capsules in Small Clear Poly Zipper Bags. Placebo Capsules Contain Glycerin Oil.

*Omega-3 group:* In the Omega-3 group, patients received Mercury-free Omega-3 Capsules containing 1000mg fish oil+EPA 180mg+DHA 120mg/ b.i.d. (Zahravi Pharm. Co Tabriz. Iran) (Figure 2).

*Placebo group:* In the placebo group, patients received Capsules containing 1000 mg of Glycerin oil/ b.i.d. (Zahravi Pharm. Co Tabriz. Iran) (Figure 2).

In both groups, all patients were blinded to the content of the capsules. 

*Outcomes: *The primary outcome was evaluating Omega-3 efficacy in improving the pure tone average. The secondary outcome was assessing Omega-3 effects on the WRS and SRT.

*Statistical analysis:* The study was designed to have 90% power to discover a fourteen dB difference (SD=17) in the pure tone average. At least 64 cases were required to obtain a conclusive primary outcome. 

ẟ=$\frac{\begin{array}{cc} \mu_{1}- & \mu_{2} \end{array}}{\sigma }$     m(size per group)=$\frac{2c}{\delta^{2}}$+1

Therefore, it was planned to include 78 participants. Because the enrollment rate was lower than expected because of the COVID-19 pandemic, the steering committee decided to prolong enrollment until December 2020. Statistical analysis was conducted utilising SPSS (version 24; IBM, Armonk, NY). Both groups' baseline characteristics were compared via Chi-square, Independent Samples Test, and the Greenhouse–Geisser. A P-value of <0.05 was considered statistically significant. 

***Minitab® 19 Statistical Software 19.2020.1 (64-bit)*** was also utilised for classification and regression tree (CART) modelling. Classification and regression trees are Nonparametric and Nonlinear. Classification and regression trees implicitly perform feature selection. It produces easy-to-understand models. The number of terminal nodes of the model was determined based on an optimal node provided by the program per se. All the quantitative and qualitative variables (i.e., PTA) were inserted to produce and predict the best model and an excellent CART. 

 Therefore, the data set was successfully split into increasingly homogenous subgroups. At each stage (node), the Gini algorithm selects an explanatory variable and divides the value with the best discrimination between two outcome classes. The CART produced an algorithm to predict the 30-day outcome of patients who had SSNHL at presentation to the hospital. The minimum number of cases for being split into internal nodes was 10, and the minimum number allowed for the terminal node was 3.

## Results

*Characteristics of the Patients*

2018 to December 2020. Thirty-three patients were randomised to the Omega-3 group and thirty-two to the placebo group. Amongst 65 patients, 41 were males (63.1%), and 24 were females (37.9%). The Omega-3 group included 22 male (66.6%) and 11 (33.4%) female patients. Nineteen cases were male (59.3%) in the placebo group, and 13 (40.7%) were female patients. Mean (S.D.) age in the Omega-3 and placebo groups was 48.1(15.37) and 44.4(13.3), respectively. There were no significant differences between both groups regarding age and sex.

Amongst all patients, 60% had hearing loss in the right ear. 47 (72.3%) patients had aural fullness and hearing loss. Thirty-three patients (50.8%) also reported acute tinnitus at the onset of hearing loss. 32.3% of patients had vertigo. Thirty-four patients (52.3%) reported underlying diseases, including diabetes (16.9%), hypertension (16.9%), cardiovascular disease (13.8%), anaemia (10.8%), hyperlipidemia (9.2%), history of upper respiratory tract infection within the last two weeks before H.L. (4.6%), reflux (9.2%), hypothyroidism (3.1%), lung diseases (3.1 %), and history of H.L. in the contralateral ear (13.8%). A history of smoking, opium addiction, and alcohol consumption was reported by 30.8, 9.2, and 3.1% of patients. 21.5% of patients also wrote about a recent journey, especially to the country's northern regions. None of the patients had a fever or systemic symptoms. 87.7% of patients had normal eardrums on examination, and 12.3% had a perforation in the tympanic membrane (Table 1).

**Table 1:** This Table Shows the Characteristics of the Patients

**N=65**		**Total** ** (%)**
Age (Mean ± S.D.)	Omega-3	48.1(15.37)
	Placebo	44.4 (13.3)
Sex	Female	24(37·9)
	Male	41(63·1)
Hearing Loss	Right	39(60)
	Left	26(40)
Aural Fullness	Negative	18(27·8)
	Positive	47(72·2)
Tinnitus	Negative	32(49·2)
	Positive	33(50·8)
Vertigo	Negative	44(67·7)
	Positive	21(32·3)
Underlying conditions	Negative	31(47·7)
	Positive	34(52·3)
Hx of Contralateral HL	Negative	56(86·2)
	Positive	9(13·8)
Hx of Smoking	Negative	45(69·2)
	Positive	20(30·8)
Alcohol Consumption	Negative	63(96·9)
	Positive	2(3·1)
Opium Addiction	Negative	59(90·8)
	Positive	6(9·2)

*Audiometric Parameters and Relationships with Symptoms and Recovery*

In the evaluation of treatment response by Modified Siegel's criteria and its relationship with each of the above variables (8), a complete response (C.R.) in pure-tone average (PTA) in frequencies of 0.5-, 1-, 2-, and 4-kHz was calculated. A final hearing level ≤ 25 dB was considered a complete recovery. Partial recovery (P.R.) was viewed as more than 15 dB hearing gain and a last hearing level of 26-45 dB. Slight improvement (S.I.) was considered more than 15 dB hearing gain and final hearing level 46-75 dB. No improvement (N.I.) when hearing gain was less than 15 dB, or the last hearing level was 76-90 dB, and Non-serviceable ear (N.S.) was the final hearing level >90 dB. 

Ultimately, a relationship between vertigo at the time of presentation to the hospital (32.3% of all patients) and C.R. was significant (P = 0.029). Amongst those patients with vertigo, only four patients showed complete recovery versus seventeen patients with no vertigo. 

Thirty-four patients at least had an underlying condition, of which only seven experienced complete recovery from hearing loss (P = 0.001). None of the other variables obtained from patients' history and findings during the examination revealed a significant relationship with the complete recovery from hearing loss.

*Imaging: *An MRI of the brain and skull base was obtained one month after the initial presentation to study central lesions. 

There were nine patients with central lesions, including three cases with a brain tumour, three cases of CP-Angle neoplasms (i.e., meningioma, vestibular schwannoma, and epidermoid), and three cases with previous evidence of ischemic changes in the temporal lobe and cerebrovascular malformations, particularly in the basilar artery. These patients were excluded from the study based on the imaging findings following approval by an expert radiologist.

*Audiometric parameters and recovery in the Omega-3 vs Placebo group*

The two groups had no significant difference before and after treatment on the average PTA at four frequencies (i.e., 0.5-, 1-, 2-, and 4-kHz bone conduction thresholds). Recovery was observed in both groups similarly (0, 10, 20, 30, and 90 days after the initial presentation).

**Fig 3:**
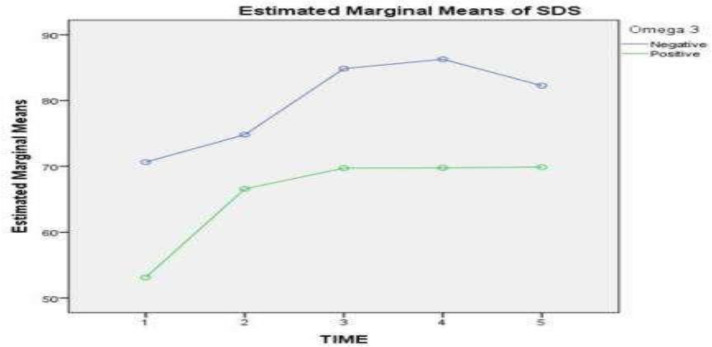
This chart shows the SDS or WRS between the placebo and omega-3 groups. As shown here, there is no difference in the recovery from SSNHL. The course of WRS changes from the beginning to the end of treatment during follow-up was significant (P = 0.001) but similar

Analysing the WRS between placebo and Omega-3 groups showed no difference in the recovery from SSNHL. In both groups, the course of WRS changes from the beginning to the end of treatment during follow-up was significant (P = 0.001), but it was similar in both groups, and there was no difference between them (P = 0.485) (Figure 3).

There was no significant difference regarding improvement between both groups in the average AC-PTA at frequencies of 0.5-, 1-, 2-, and 3-kHz. The 3-kHz threshold was obtained by averaging the thresholds at 2 and 4 kHz based on Richard K. Gurgel and associates (9). In both groups, the AC-PTA average at frequencies of 0.5-, 1-, 2-, and 3-kHz from the beginning to the end of treatment was significant (P = 0.000). However, the recovery course was similar in both groups, with no significant difference (P = 0.557).In the assessment of SRT between both groups, results revealed no difference regarding the recovery.

**Fig 4:**
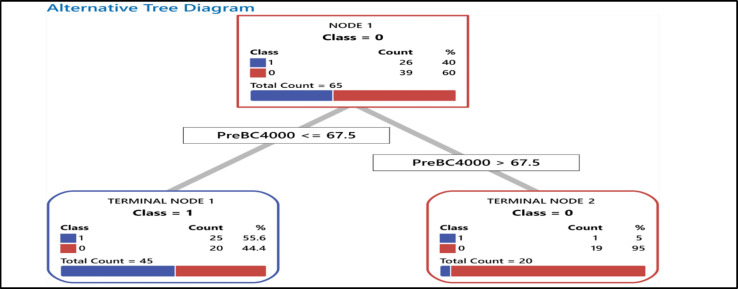
This algorithm outlines recovery from SSNHL following the treatment. In total, 26 patients (40%) had a complete response to treatment (final hearing level ≤25 dB) and a full recovery from hearing loss. Patients with a BC-PTA threshold of ≤ 67.5 in 4-kHz showed a more remarkable recovery threshold of>67.5(55.6%). The blue colour represents the complete response

*Total Recovery : *Twenty-six patients (40%) had a complete response to treatment, and 4.6% and 6.2% had P.R. and S.R., respectively —50.8% recovery from SSNHL. Ultimately, patients with hearing loss with a B.C. threshold≤ of 67.5 in 4-kHz showed higher recovery than those with a BC-PTA threshold> 67.5 —55.6% versus 5 % (Figure 4). Other frequencies did not have meaningful results. CART showed this cutoff as a significant one after the treatment. 

## Discussion

Idiopathic sudden sensorineural hearing loss (SSNHL) has been a complex diagnosis for many years and requires excluding many underlying factors to confirm the diagnosis. Some studies have proposed vascular events due to the temporal course similar to the disease progression (4,10,11). This theory failed to reveal the relationship between this phenomenon and vascular events (12). The standard treatment for this disease is the steroid, either orally or through intratympanic injection (1,13,14).Nowadays, many pharmaceutical companies have revenue from marketing these supplements (i.e., Omega-3). Numerous studies have shown the benefits of Omega-3 in improving cardiovascular function, autoimmune diseases, lipid profile, acute diseases, and preventing hearing loss (5,6,15-19). The question is whether these supplements are beneficial to improving these diseases or not. On the other hand, vascular disorders and inflammation in the inner ear have been stated as the aetiologies of SSNHL.

In the current study, SSNHL was observed in 60% of male patients, and most of them (in both Omega-3 and placebo groups) were 40-60 years old. In previous studies, SSNHL has been observed in the same age range. However, regarding sex, in some studies, patients were frequently male, and some were female (14, 20). In the current study, a history of sudden H.L. or deafness was observed in all patients, and 72.2% of patients complained of a sudden feeling of fullness in the ear (aural fullness). This finding is also evident in previous studies (1). In the present study, approximately 51% of patients had treatment response and recovery from SSNHL. In a study by Cheng et al. (8), 50% of 110 patients who received oral steroid therapy and I.T. injections —in combination — showed recovery from hearing loss. The treatment generally improves half of the patients who come to medical attention with SSNHL. In 2021, Marie N Shimanuki et al.(21) revealed that age, the threshold at the onset of hearing loss, and vertigo were predictors of hearing recovery from SSNHL. In the current study, patients with underlying conditions or vertigo intercurrent to SSNHL did not recover completely. Age and other factors did not affect the recovery from the disease. Therefore, patients who do not have vertigo at the initial presentation and do not have an underlying condition are more likely to recover from SSNHL. A study by Maggie Kuhn et al.(22) on prognostic factors in the recovery from SSNHL demonstrated that vertigo, hearing loss at higher frequencies, failure to visit a doctor within the initial two weeks, and underlying conditions had negatively impacted the recovery. In the current study, patients with hearing loss at a frequency of 4-kHz and BC-PTA> 67.5 had less response to the steroid, and the hearing results were lower in these patients following the treatment. This finding was consistent with a study by Da Jung Jung et al.(14), in which patients with hearing loss and PTA ≥ 70 dB had lower hearing outcomes. This relationship has been mentioned in the literature also (1).In some studies, the efficacy of Omega-3 was significant in improving vascular function, acute diseases and decreasing inflammatory phases (5,19,23). 

However, this supplement did not enhance vascular function (24,25). The efficacy of Omega-3 adjunctive therapy in SSNHL was investigated for the first time in the current study. This supplement did not have a significant effect on the recovery from SSNHL. Gopinath and colleagues (6) concluded that this supplement positively affected hearing and presbycusis progression. In this study, researchers presumed that higher intakes of long-chain n−3 PUFAs and regular weekly fish consumption prevent or delay the development of age-related H.L. It is still unexplained what this positive effect is and how it plays an essential protective factor. In the current study, this supplement as adjunctive therapy was ineffective in improving hearing in SSNHL patients, and there was no therapeutic effect for this supplement. 

Although it was not statistically significant, patients in the Omega-3 group had faster recovery than placebo. As Teresa Partearroyo and colleagues (26) reported, different human hereditary rare conditions are related to homocysteine (Hcy) metabolism, particularly SNHL. Hyperhomocysteinemia has revealed a definite correlation with the progress of neurological dysfunctions, chronic kidney disease, osteoporosis, gastrointestinal disorders, cancer, and some congenital deficiencies (27). 

In Teresa Partearroyo's study, researchers stated that long-term dietary supplementation with omega-3 has protective effects by enhancing Hcy metabolism, cell survival, and hearing acuity. This supplement may support cell junctions by inhibiting Hcy accumulation, harming the Cochlea’s hearing receptors. Based on the mentioned points, a sudden onset of SSNHL may be due to metabolic, inflammatory, or vascular insults in the labyrinth or near the auditory nerve. An underlying condition might be an initiating factor for the onset of SSNHL. All steroid-based treatment modalities only perceive recovery in 50% of SSNHL patients. Why vertigo is an adverse prognostic factor in the recovery from SSNHL is still unexplored. However, this symptom may prove the involvement of the vestibular components—the semicircular canals or vestibular nerve— in the labyrinth. In further studies, Vertigo in SSNHL might be categorised as a distinct subgroup in these patients. In later studies, researchers may understand the main potential aetiologies to concentrate on treating this disease with a multidisciplinary approach.

## Conclusions

Many therapies have been used as adjunctive therapies with steroids in treating SSNHL patients. This study investigated the efficacy of Omega-3 in these patients for the first time. The results revealed no notable difference between Omega-3 recipients and the placebo group regarding the recovery from SSNHL. Moreover, complete recovery occurs in half of these patients. Vertigo and underlying conditions are intercurrent with hearing loss, which would negatively impact the recovery.

*Methodological considerations/limitations*

First, the absolute effects of Omega-3 on SSNHL patients could not be clarified because, ethically, ignoring the standard treatment—Steroids— for these patients is not logical. Therefore, this traditional treatment may conceal some effects of this supplement on treatment. Second, because of the treatment protocol for SSNHL, researchers of the current study had to replace the treatment with an oral steroid with an I.T. steroid when there was no response after ten days. Therefore, this issue may harm the treatment response with Omega-3. To resolve this problem, patients with contraindications for oral steroids were excluded to decrease this negative effect on treatment.
